# Renal and cardiac biopsy findings in an adolescent patient with the 3243A>G mitochondrial DNA mutation: Favorable renal prognosis post renal transplantation from the mother 

**DOI:** 10.5414/CNCS111422

**Published:** 2024-06-25

**Authors:** Hisashi Kamido, Shigekazu Kurihara, Yuki Oba, Masayuki Yamanouchi, Tatsuya Suwabe, Katsuyuki Miki, Yuki Nakamura, Yasuo Ishi, Kei Kono, Kenichi Ohashi, Naoki Sawa, Yoshifumi Ubara

**Affiliations:** 1 Nephrology Center, Toranomon Hospital, Minato,; 2 Department of Pathology, Toranomon Hospital, Tokyo, and; 3 Department of Human Pathology, Tokyo Medical Dental University, Tokyo, Japan

**Keywords:** mitochondrial DNA mutation 3243A>G, mitochondrial nephropathy, mitochondrial cardiomyopathy, mitochondrial encephalopathy, renal transplantation

## Abstract

We investigated the pathogenesis of a perihilar variant of focal segmental glomerulosclerosis detected by kidney biopsy in a 16-year-old male. The disease was refractory to steroid therapy, and at the second kidney biopsy, abnormal mitochondrial proliferation was newly observed in the podocytes. The patient also developed late-onset hearing loss and had a family history of diabetes, and genetic testing confirmed the mitochondrial DNA mutation 3243A>G (48%). Eight months after hemodialysis was started, encephalopathy occurred presumably due to rapid dehydration. After changing dialysis into continuous ambulatory peritoneal dialysis, encephalopathy was resolved, but the patient developed myocardial hypertrophy, probably because of the myocardial overreaction to congestion. A myocardial biopsy showed mitochondrial proliferation in the myocardium. After renal transplantation from his mother with a heteroplasmy of 4%, the cardiomyopathy improved, and the renal function has remained stable for 4 years. We speculated that the abnormal mitochondrial morphology in the kidney and heart may be characteristic of mitochondrial genetic disease, and renal transplantation from the mother with a low heteroplasmy was considered desirable for mitochondrial nephropathy with poor prognosis.

## Introduction 

Patients with focal segmental glomerulosclerosis (FSGS) often exhibit resistance to steroids, leading to an unfavorable prognosis that may necessitate kidney transplantation. Recent findings indicate the identification of genetic disorders in cases of FSGS [1, 2, 3] and mitochondrial disorders should be considered, especially in cases with a family history of diabetes and hearing impairment [4]. 

We experienced a case of FSGS whose diagnosis of mitochondrial nephropathy was delayed due to the postponed onset of hearing loss. 

## Case report 

**Figure 1:**
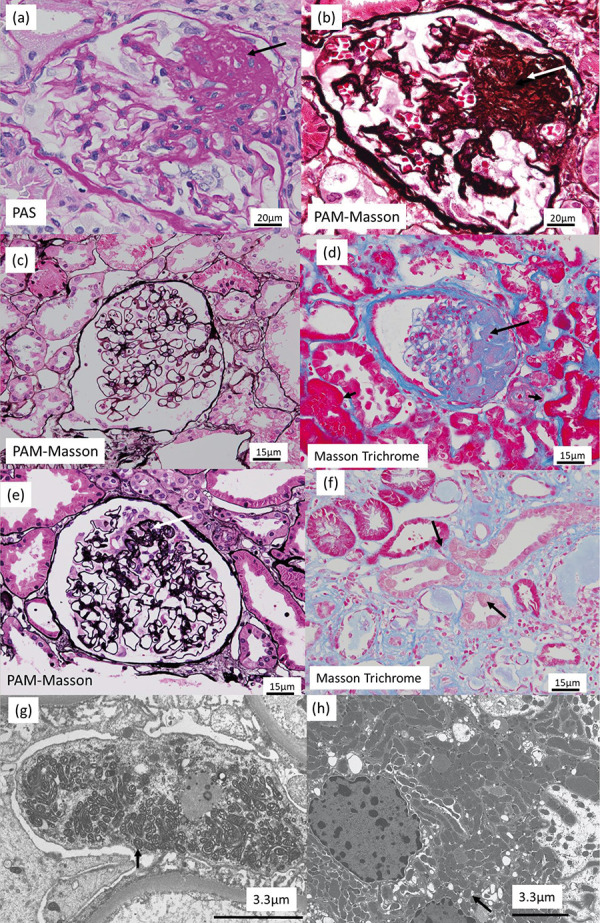
Kidney biopsy findings. a, b: The first kidney biopsy showed sclerotic lesions in the glomerular capillaries (arrow) near the vascular pole, and perihilar variant of focal segmental glomerulosclerosis was diagnosed (original magnification × 400). a: periodic acid Schiff stain; b: periodic acid methenamine silver (PAM)-Masson stain. c: PAM-Masson stain showed no significant findings in the other glomeruli (original magnification × 200). d: One glomerulus showed a severe sclerotic lesion with hyalinization (arrow) in the glomerular capillaries near the vascular pole (Masson trichrome stain; original magnification × 200). Masson trichrome staining showed strong red staining (small arrow) in the proximal tubules. e: One glomerulus showed partial sclerosis and epithelial cell proliferation (large arrow; PAM-Masson stain; original magnification × 200). f: Granular swollen epithelial cells (arrow), defined by enlarged tubular epithelial cells with coarse granules, were found in some distal tubules and collecting ducts (original magnification × 200). g: Electron microscopy revealed abnormal mitochondrial proliferation (arrow) in the podocytes. h: Electron microscopy revealed abnormal mitochondrial proliferation (arrow) in the proximal tubular epithelium.

The 16-year-old male patient underwent a first kidney biopsy because his serum creatinine (Cr) level was 0.73 mg/dL and urinary protein excretion (UP) was 0.6 g/day. 13 glomeruli were obtained; none was sclerosing, but 1 showed sclerotic lesions of the glomerular capillaries near the vascular pole (Figure 1a, b); there were no significant findings in the other glomeruli (Figure 1c). An immunofluorescence analysis showed no significant deposition of immunoglobulin (Ig) G, IgA, C3, C4, or C1q. Electron microscopy showed foot process effacement but no electron-dense deposits. Perihilar variant of FSGS was diagnosed, and prednisolone (PSL) 30 mg/day was started. However, PSL had little effect, and was reduced to 10 mg/day. Instead, mizoribine 150 mg/day was added. 

At age 18, UP increased to 4.0 g/day, and additional treatment with cyclosporin 100 mg/day was started. At age 21, UP increased to 6.0 g/day and Cre progressed to 2.08 mg/dL, and a second kidney biopsy was performed. Of the 16 glomeruli obtained, 9 showed global sclerosis, 1 had a severe sclerotic lesion with hyalinization in the glomerular capillaries near the vascular pole (Figure 1d), and 1 had partial sclerosis and epithelial cell proliferation (Figure 1e). The findings confirmed the diagnosis of perihilar variant of FSGS. The patient was treated with PSL 50 mg/day, and low-density lipoprotein apheresis was performed. However, the treatment had insufficient effect, and the patient was admitted to our hospital. 

On admission, the patient was 170 cm tall, weighed 56 kg, and had a body mass index of 19.4 kg/m^2^. His blood pressure was 139/96 mmHg, and heart rate, 89 bpm. Laboratory test results were as follows; albumin, 4.2 mg/dL; blood urea nitrogen, 70 mg/dL; Cre, 4.97 mg/dL; estimated glomerular filtration rate (eGFR), 13.8 mL/min/1.73m^2^; glycosylated hemoglobin, 6.0%; cystatin C, 3.58 mg/L; IgG, 728 mg/dL; IgA, 152 mg/dL; IgM, 69 mg/dL; C3 complement, 114 mg/dL; C4 complement, 40 mg/dL; CH50, 59 U/mL; antinuclear antibody, negative; and UP, 3.9 g/day. The urine sediment contained less than 1 red cell per high-power field. 

### Diagnosis 

Facing treatment-resistant FSGS at a young age necessitates consideration of a potential genetic disorder. First, a detailed family history interview was obtained: The mother’s sister and 2 brothers had diabetes, but there was no family history of nephropathy. The admitting physician encountered challenges in communication with the patient during the interview, despite the patient’s normal educational background, leading to suspicions of a potential hearing impairment. Further inquiry revealed that the patient had indeed been experiencing a progressive loss of hearing, and audiometry showed bilateral moderate sensorineural hearing loss in the high-frequency range (right ear, 37.5 dB; left ear, 33.8 dB). 

Second, a panel genetic analysis covering 38 causative genes for hereditary FSGS was performed on all exons and exon-intron boundary regions by next-generation sequencing. However, all tests were negative. Subsequent mitochondrial genetic testing was performed with peripheral blood and showed mitochondrial DNA mutation 3243A>G (48%). 

Then, we gathered information specific to mitochondrial diseases. The patient exhibited pain in both lower extremities, decreased skin perspiration, and tinnitus. Chest X-ray showed an enlarged cardiothoracic ratio (CTR) of 61%, and echocardiography revealed left ventricular hypertrophy with an ejection fraction of 73% and a left ventricular mass index (LVMI) of 123 g/m^2^, with no observed abnormal wall motion. Electrocardiogram showed Wolff-Parkinson-White syndrome. Peripheral nerve motor examinations indicated suspected peroneal and tibial nerve axonopathy. Serum lactic acid was 56 mg/dL (reference range, 4.2 – 17.0 mg/dL), and pyruvic acid was 2.0 mg/dL (reference range, 0.3 – 0.9 mg/dL). On the basis of these findings, FSGS was presumed to be due to mitochondrial nephropathy. 

When we re-examined the light microscopy images of the kidney, we noticed that Masson trichrome staining showed strong, red staining in the proximal tubules (Figure 1d), a feature not usually seen in kidney biopsies from patients with end-stage renal failure. The staining was stronger in the second biopsy, probably because of increased mitochondrial activity. Granular swollen epithelial cells, which have been reported as a characteristic finding in mitochondrial nephropathy [5], were found in some distal tubules and collecting ducts (Figure 1f). Electron microscopy revealed abnormal mitochondrial proliferation in the podocytes (Figure 1g) and partial foot process effacement. Abnormal mitochondrial proliferation was also seen in the proximal tubular epithelium (Figure 1h). Thus, mitochondrial nephropathy was confirmed both genetically and pathologically. 

### Treatment of end-stage renal disease 

**Figure 2:**
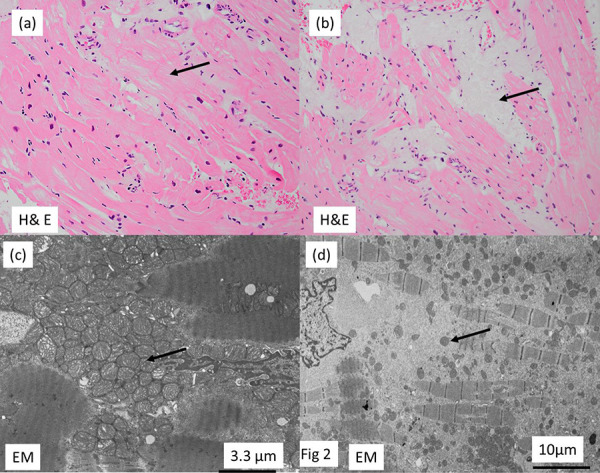
Myocardial biopsy. a: Light microscopy of the myocardial biopsy showed muscle fibers with indistinct borders and somewhat lucent areas (arrow; hematoxylin and eosin (H & E) stain). b: Vacuolated muscle fibers areas (arrow) were seen (H & E stain). c: Electron microscopy showed abnormal mitochondrial proliferation between the fragmented muscle fiber bundles. d: In vacuolated muscle fiber areas, there was a decrease in the number of mitochondria (arrow) and muscle fibers.

Three months after admission, hemodialysis (HD) was started with 3 times a week for 4 hours per session. Eight months after the initiation of HD, the patient experienced seizures characterized by loss of consciousness, facial, and limb spasms following the completion of HD. The symptoms showed improvement the next day. Electroencephalography and magnetic resonance imaging showed no abnormalities. Serum ammonia levels were measured to rule out the possibility of portal systemic encephalopathy [6] and found to be normal. In addition, lactate and pyruvate were measured before and after HD, but no significant increase was found. Post-HD seizures occurred only when more than 2 – 3 kg of fluid was removed by a single HD, and no further pathophysiology could be identified. The cause of the encephalopathy was presumed to be rapid dehydration, and the dialysis was changed to CAPD. CAPD prevented the development of encephalopathy but resulted in poor fluid removal. The patient developed cardiac hypertrophy and pericardial effusions (LVMI, 162.9 g/m^2^, and CTR, 72%). A myocardial biopsy was performed. Light microscopy showed muscle fibers with indistinct borders and somewhat lucent areas (Figure 2a) and vacuolated muscle fibers areas (Figure 2b). Electron microscopy showed abnormal mitochondrial proliferation between the fragmented muscle fiber bundles (Figure 2c). In the vacuolated muscle fiber areas, the number of mitochondria and muscle fibers was decreased (Figure 2d). CAPD alone was not sufficient, and weekly HD was added. 

### Renal transplantation as the next treatment option 

The patient’s father was diagnosed with a blood disorder, while the patient’s mother possessed the mitochondrial DNA mutation 3243A>G with a low heteroplasmy rate of 4%. Despite carrying this mutation, she maintained normal renal function and urinary protein, and exhibited no symptoms of diabetes or hearing impairment. Consequently, she was deemed a donor for the renal transplantation. Following surgery, the patient demonstrated improved renal function (Cr, 1.7 mg/dL; eGFR, 39.0 mL/min/1.73m^2^), accompanied by a weight loss of 5 kg. At 1 year follow-up, CTR and LVMI had recovered to 53% and 125.2 g/m^2^, respectively. Four years post-surgery, renal function has remained stable (Cr, 1.9 mg/dL; eGFR, 39.0 mL/min/1.73m^2^), and a rejection has not been observed. 

## Discussion 

We experienced a case of FSGS whose diagnosis of mitochondrial nephropathy was delayed due to the postponed onset of hearing loss. 

In cases of mitochondrial nephropathy, previous reports have identified segmental sclerosis and hyalinosis near the vascular pole as characteristic features [7, 8]. In this case, both renal biopsies showed the perihilar variant of FSGS, commonly known in association with obesity-related kidney disease [9]. This variant may also frequently occur in mitochondrial disorders. In the same previous reports, electron microscopy has been known to reveal a marked increase in the number of abnormal mitochondria with various shapes, sizes, and disarrangement of cristae in podocytes and glomerular epithelium [7, 8]. In our case, mitochondrial proliferation in podocytes and proximal tubules was not seen in the first biopsy and only became apparent in the second biopsy. This may indicate an overreaction of mitochondria in the remaining renal tissue as renal failure progresses. 

Ochiai et al. [10] reported that characteristic findings in mitochondrial cardiomyopathy are an increased number of mitochondria of variable sizes and abnormal mitochondrial cristae. In our case, we also observed areas with same characteristics, and vacuolated areas that were deficient in both myocardial fibers and mitochondria. The myocardial overreaction to congestion may have manifested itself in the form of abnormal mitochondrial proliferation in the myocardium. 

Several reports have highlighted the relationship between heteroplasmy and disease activity. Concerning renal involvement, a higher percentage of heteroplasmy is associated with a worse renal prognosis [11]. The median heteroplasmy in patients with the mitochondria DNA 3243A>G mutation undergoing renal transplantation is reported to be 24% [12]. The heteroplasmy rate of 48% in our case is higher, which aligns with the poor renal prognosis. However, maternal heteroplasmy was observed to be as low as 4%, indicating a favorable renal prognosis. Notably, renal transplantation demonstrated the maintenance of stable renal function for a minimum of 4 years. 

In summary, we experienced a patient with FSGS developing hearing loss during the course of the disease. The patient subsequently developed encephalopathy, presumably due to rapid dehydration in HD, and myocardial hypertrophy, probably because of the myocardial overreaction to congestion in CAPD. As a result, renal transplantation was considered desirable for mitochondrial nephropathy with poor prognosis. 

## Availability of data and materials 

The dataset supporting the conclusions of this article is included within the article. 

## Ethics approval and consent to participate 

This case report was written in compliance with the Declaration of Helsinki. 

## Consent for publication 

Written consent for publication of this case report was obtained from the patient. 

## Authors’ contributions 

HK performed the primary manuscript preparation; HK and YU wrote the paper. HK had primary responsibility for the final content. HK, SK, YO, MY, TS, KM, YN, YI, KK, KO, NS, and YU reviewed the paper and revised it critically. All authors have read and approved the final manuscript. 

## Funding 

None. 

## Conflict of interest 

The authors declare no competing financial interest and no conflict of interest.
